# A-type K^+^ channels impede supralinear summation of clustered glutamatergic inputs in layer 3 neocortical pyramidal neurons

**DOI:** 10.1016/j.neuropharm.2018.07.005

**Published:** 2018-09-15

**Authors:** Ágota A. Biró, Antoine Brémaud, Joanne Falck, Arnaud J. Ruiz

**Affiliations:** UCL School of Pharmacy, Brunswick Square, London WC1N 1AX, United Kingdom

**Keywords:** I_A_, Mice, Neocortex, RuBi-glutamate, Phrixotoxin-2, 4-Aminopyridine

## Abstract

A-type K^+^ channels restrain the spread of incoming signals in tufted and apical dendrites of pyramidal neurons resulting in strong compartmentalization. However, the exact subunit composition and functional significance of K^+^ channels expressed in small diameter proximal dendrites remain poorly understood. We focus on A-type K^+^ channels expressed in basal and oblique dendrites of cortical layer 3 pyramidal neurons, in *ex vivo* brain slices from young adult mice. Blocking putative Kv4 subunits with phrixotoxin-2 enhances depolarizing potentials elicited by uncaging RuBi-glutamate at single dendritic spines. A concentration of 4-aminopyridine reported to block Kv1 has no effect on such responses. 4-aminopyridine and phrixotoxin-2 increase supralinear summation of glutamatergic potentials evoked by synchronous activation of clustered spines. The effect of 4-aminopyridine on glutamate responses is simulated in a computational model where the dendritic A-type conductance is distributed homogeneously or in a linear density gradient. Thus, putative Kv4-containing channels depress excitatory inputs at single synapses. The additional recruitment of Kv1 subunits might require the synchronous activation of multiple inputs to regulate the gain of signal integration.

## Highlights

• We focus on A-type K+ channels expressed in oblique and basal dendrites.
• Putative Kv4 subunits depress excitatory signals generated by single spine excitation.
• Kv4 and Kv1 regulate supralinear signal integration at clustered dendritic spines.
• A computational model simulates Kv-mediated modulation of dendritic integration.

## 1 Introduction

Dendritic integration of synaptic potentials is regulated by several biophysical and molecular factors linked to synaptic transmission and changes in intrinsic excitability (Araya, 2014; Branco and Hausser, 2010; Major et al., 2013; Spruston, 2008; Stuart and Spruston, 2015; Williams and Stuart, 2002). These factors include the number, location, timing, and sequence of operation of synaptic inputs (Katz et al., 2009; Magee, 2000). Signal integration is also dependent on the electrotonic structure of the dendritic tree (Korogod and Tyc-Dumont, 2009), as well as the nature, density, and gradient of voltage-gated ion channels present in somato-dendritic membranes (Hille, 2001).

Amongst channels that regulate dendritic integration, Kv1 and Kv4 pore-forming subunits are strongly expressed in rodent neocortical pyramidal neurons (Burkhalter et al., 2006; Carrasquillo et al., 2012; Guan et al., 2011; Norris and Nerbonne, 2010) where they mediate a transient somato-dendritic A-type K^+^ current (I_A_). Kv1.4, Kv4.2 and Kv4.3 can produce an A-type current when combined with the proper Kv beta subunit (Jerng et al., 2004; Marionneau et al., 2009; Ovsepian et al., 2013). Electron microscopy analysis has also revealed that Kv1.4, Kv4.2 and Kv4.3 subunits localize in dendritic spines and shafts (Burkhalter et al., 2006; Juiz et al., 2000; Kerti et al., 2012). Such preferential localization of I_A_ channels combined with a subthreshold activation range and fast activation kinetics is thought to dampen excessive depolarization caused by strong synaptic excitation. In addition, neocortical pyramidal neurons exhibit a persistent and slow-activating component to outward K^+^ currents (Bekkers, 2000b; Foehring and Surmeier, 1993; Guan et al., 2006, 2007, Korngreen and Sakmann, 2000; Locke and Nerbonne, 1997), which is mediated by Kv1 and Kv2 channels (Bekkers and Delaney, 2001; Bishop et al., 2015; Guan et al., 2006, 2007, 2015; Pathak et al., 2016).

Dendritic patch-clamp recordings from CA1 pyramidal neurons have revealed a functional six-fold increase in I_A_ current density along the proximal to distal path of the main apical dendrite (Hoffman et al., 1997). This distribution in positive gradient restrains dendritic plateau potentials and reduces the amplitude of back-propagating axo-somatic action potentials (Andrasfalvy et al., 2008; Cai et al., 2004) enabling synaptic plasticity and branch-specific storage (Chen et al., 2006, 2011; Frick et al., 2003; Golding et al., 1999; Losonczy and Magee, 2006; Losonczy et al., 2008). Similarly, an increasing density of dendritic Kv4 channels along the apical dendrite is maintained by the auxiliary subunit DPP6, restricting action potential back-propagation and calcium spike generation (Sun et al., 2011). In contrast, a small decrease in the density of dendritic K^+^ channels has been reported in the large dendrite of layer 5 (L5) pyramidal neurons in young rats (Korngreen and Sakmann, 2000).

Despite this wealth of evidence, much less is known about A-type K^+^ channels in small diameter neocortical dendrites. I_A_ regulates input summation throughout the apical dendritic tuft of L5 pyramidal neurons resulting in strong electrical compartmentalization at distal synapses (Harnett et al., 2013). However, it remains unclear whether such I_A_-dependent regulation of signal integration occurs in basal and oblique dendrites of layer 3 (L3) pyramidal neurons. In addition, novel computational approaches are needed to comprehend the functional significance of I_A_ in small diameter dendrites, the vast majority of which is unamenable to patch-clamp recordings.

To understand how K^+^ channels regulate signal integration in L3 pyramidal neurons, we examine the effect of K^+^ channel blockers on electrical membrane properties and summation of excitatory synaptic potentials. We use two-photon laser scanning microscopy and photolysis of RuBi-glutamate to mimic the activation of excitatory synapses in basal and oblique dendrites. We show that blocking putative Kv4 channels enhances the response evoked by glutamate uncaging at a single dendritic spine. The block of putative Kv1 channels has no effect on such unitary responses. Both Kv1 and Kv4 channels impede supralinear summation of glutamatergic signals elicited by synchronous activation of clustered spines. Using computational modeling we show that a linear gradient in A-type conductance density distribution along the dendrite is not required to explain the effect of K^+^ blockers on dendritic integration. Remarkably in these neurons the interplay between A-type K^+^ channels and NMDA receptors results in a highly dynamic modulation of the gain of dendritic integration of glutamatergic signals.

## 2 Results

### 2.1 Physiological and pharmacological properties of EPSPs evoked by glutamate uncaging at single spines in basal and apical oblique dendrites

**Fig. 1:**
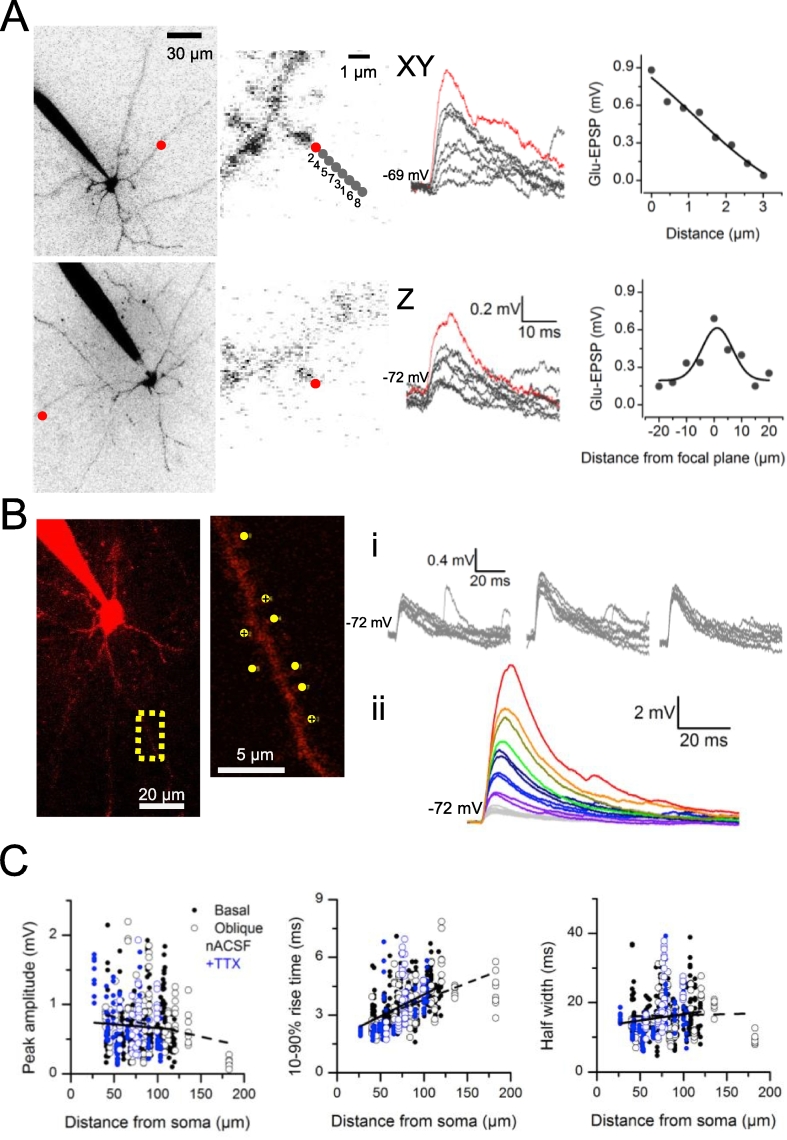
**Glutamate uncaging at single and multiple dendritic spines imaged with 2-photon laser scanning microscopy in L3 pyramidal neurons. *A***, 3-D resolution of single-photon uncaging at single dendritic spines and corresponding somatic gluEPSPs. Left, maximum intensity projection of 2-photon images of two Alexa Fluor 594 (150 μM) filled L3 pyramidal neurons. Single spines were chosen randomly along the basal dendrites (at 50 μm, top; and 100 μm from the soma, bottom cell). Top, moving the uncaging beam in random order (numbered spots, 1–8) along a longitudinal axis within the focal plane of the spine head, elicits EPSPs of graded amplitudes, the largest of which is obtained when the beam is positioned closest to the spine head (red spot; corresponding voltage trace is red). Bottom, positioning the uncaging beam along the Z-axis at different distances from the spine head (red spot, in focus) yields graded EPSPs. The amplitude of gluEPSPs decays almost linearly with distance from the spine head in the focal plane (top right; double Boltzmann fitting). Uncaging at different focal planes yields a bell-shaped relation (bottom right; Gauss fitting). ***B***, Properties of gluEPSPs elicited by uncaging at single spines and summation when uncaging at several spines. Maximum intensity projection of a 2-photon Z-stack from an Alexa Fluor 594-filled neuron with uncaging region of interest magnified showing 8 individual uncaging sites. (i) Superimposed gluEPSPs evoked by uncaging at single spines (3 different spines, the 3 crossed-over yellow spots; 8–9 successive trials for each spine). No run-down is observed. (ii) Co-stimulation of an increasing number of spines evokes gluEPSPs of graded amplitudes. Color traces (single traces or averages of 2 traces) correspond to gluEPSPs elicited at 1–8 spines, as indicated on the imaged dendrite. Grey is for single sites, same colors indicate same number (2, 3 or 4) but different combination of co-stimulated spines. ***C***, The amplitude of gluEPSPs evoked by uncaging at single spines does not relate to distance from the soma (<60 μm: 0.73 ± 0.40 mV, n = 136 spines; 60–90 μm: 0.67 ± 0.39 mV, n = 208 spines; > 90 μm: 0.70 ± 0.40 mV, n = 160, Kruskal-Wallis test, *P* = 0.320), nor does the half width (right). The rise time is increased with distance both for oblique and basal dendrites (<60 μm: 2.68 ± 0.86 ms, n = 135 spines; 60–90 μm: 3.64 ± 1.03 ms, n = 202 spines; > 90 μm: 4.16 ± 1.02 ms, n = 154 spines; Kruskal-Wallis test, *P* = 2.69E^−34^). Linear fit: obliques (dashed) and basals (solid). Grey and black symbols, recordings in nACSF; blue symbols, in TTX (1 μM). Closed symbols, spines on basal branches; open symbols, spines on obliques. Average uncaging distance from the soma was 83.1 ± 27.5 μm; average span of 8 spines, 23.4 ± 6.7 μm, n = 63 dendrites; yielding an average distance between stimulated spines of 3.3 ± 1.0 μm. (For interpretation of the references to colour in this figure legend, the reader is referred to the web version of this article.)

We recorded from the soma of L3 pyramidal neurons in current-clamp mode at 32 °C with pipettes containing Alexa Fluor 594 (150 μM), and imaged portions of basal and apical oblique dendrites using two-photon laser scanning microscopy (distance from soma: 27–183 μm). We selected dendritic segments that showed no overlap with neighboring dendrites from the same neuron above or below the focal plane. Glutamate receptors on individual spines were stimulated using 0.2 ms single-photon uncaging of bath-applied RuBi-glutamate (500 μM). The resulting glutamate-evoked EPSPs (gluEPSPs) were detected at somatic level. The lateral resolution of glutamate uncaging was near 3 μm for ∼0.9 mV responses whilst uncaging at positions located 10 μm away from the focal plane of the spine head halved gluEPSPs (Fig. 1A). Gradually increasing laser intensity enhanced the peak and half width of gluEPSPs, and repeatedly uncaging at the same spine head every 15 s yielded stable and reproducible responses indicating little phototoxicity (Fig. 1B). In normal artificial cerebro spinal fluid (nACSF) the mean peak amplitude of gluEPSPs elicited by uncaging at single spines in basal dendrites was similar to that of gluEPSPs evoked in apical obliques (medians; oblique: 0.88 mV, n = 168 spines in 13 dendrites; basals: 0.87, n = 96 spines in 7 dendrites; *P* = 0.48, Kruskal-Wallis test). The distribution of peak amplitude of gluEPSPs recorded in the presence of tetrodotoxin (TTX 1 μM; mean ± S.D., 0.7 ± 0.38 mV; n = 152 spines in 19 dendrites) was unimodal and not different from that of gluEPSPs recorded in nACSF (0.69 ± 0.40 mV, 352 spines from 44 dendrites; *P* = 0.77, unpaired *t*-test). Glu-EPSP amplitude remained constant with increasing distance from the soma although the increased rise time indicated cable filtering in both basal and apical oblique dendrites (Fig. 1C). Such ranges of amplitude and rise time were comparable to those reported for gluEPSPs elicited by 2-photon uncaging at single spines in brain slices, in L3 neurons (Branco and Hausser, 2011), hippocampal pyramidal cells (Losonczy and Magee, 2006; Makara et al., 2009) or dentate granule cells (Krueppel et al., 2011).

### 2.2 A-type K^+^ channels depress gluEPSPs generated by single spine activation

**Fig. 2:**
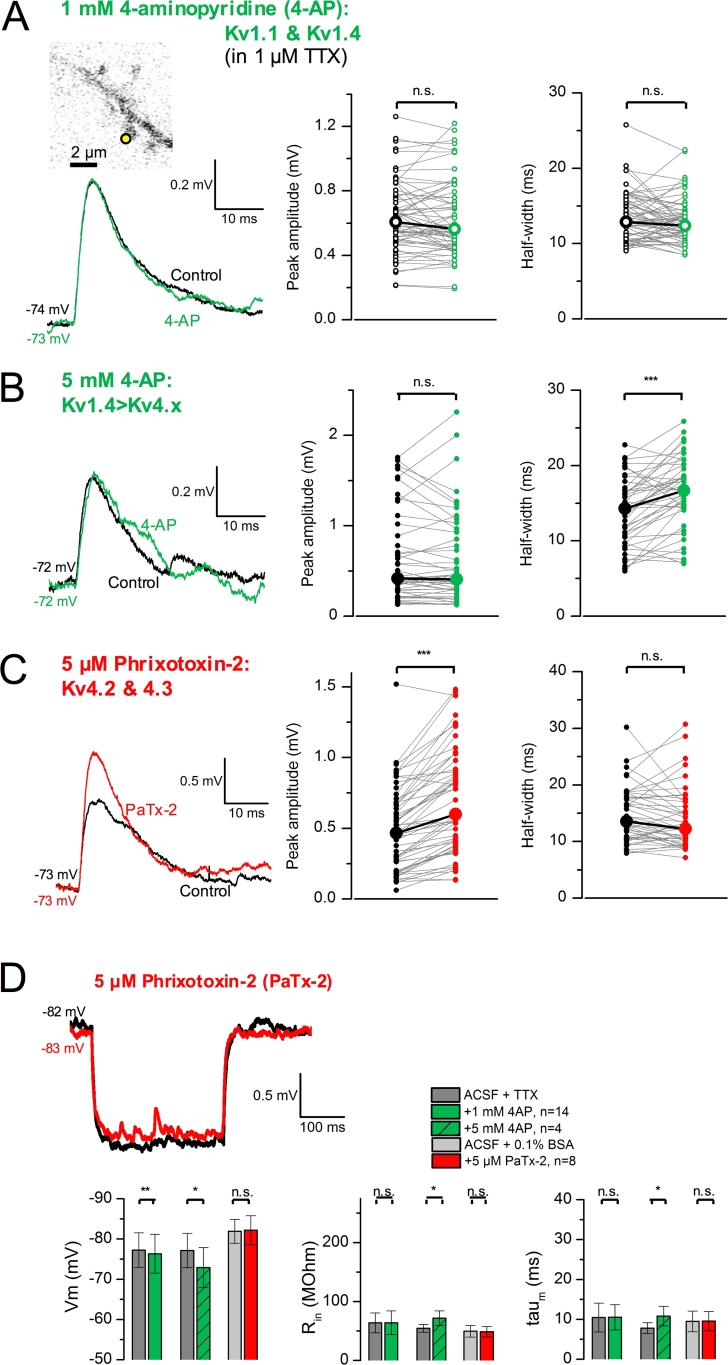
**Blocking various K**^**+**^**channels has differential effects on gluEPSPs generated by single spine activation. *A***, Superimposed gluEPSPs (average of 21 consecutive trials) evoked by uncaging at a single spine (inset, yellow spot) showing no-effect of 1 mM 4-AP. Summary plots showing no change in gluEPSP characteristics (medians; peak amplitude: 0.61 *versus* 0.56 mV, *P* = 0.466; half width: 12.90 *versus* 12.37 ms, *P* = 0.141; paired *t*-tests, data are from 64 spines, 8 dendrites in 8 neurons). ***B***, Superfusion of 4-AP (5 mM) enhances the duration but not the amplitude of gluEPSPs evoked by uncaging at single spines (traces average of 8 consecutive trials). Summary data are from 48 spines, 6 dendrites in 5 neurons (peak amplitude: 0.42 *versus* 0.41 mV, Wilcoxon signed rank tests, *P* = 0.246; half width: 14.31 *versus* 16.69 ms, paired *t*-test, *P* = 9E^−6^). ***C***, Superimposed traces show gluEPSPs evoked by uncaging at a single spine (averages of 3 consecutive trials), in control condition (0.1% BSA, black), and in the presence of phrixotoxin-2 (PaTx-2, 5 μM, red). In population data, the peak amplitude increases (0.47 mV *versus* 0.60 mV, Wilcoxon signed rank test, *P* = 3.E^−10^), whereas the half width does not change (13.52 *versus* 12.22 ms, *P* = 0.157; data are from 56 spines, 7 dendrites, 6 neurons). ***D***, Bar graphs summarizing the effects of K^+^ channel blockers on passive electrical membrane properties. All experiments involving 4-AP were conducted with TTX (1 μM) included in the perfusion solution, and those involving phrixotoxin-2 had 0.1% BSA. Data are from 4 to 14 neurons. (For interpretation of the references to colour in this figure legend, the reader is referred to the web version of this article.)

To get insight into the nature of K^+^ conductances present in basal and oblique dendrites, we focused on pharmacological compounds that have been reported to inhibit A-type K^+^ channels in cortical slice preparations (Kang et al., 2000; Kim et al., 2007; Korngreen and Sakmann, 2000; Makara et al., 2009; Pathak et al., 2016; Wang et al., 2014; Zhou and Hablitz, 1996). TTX (1 μM) was added to the control perfusion solution to prevent epileptiform activity. We started by examining the effect of low millimolar 4-aminopyridine (4-AP, 1 mM) on gluEPSPs evoked by uncaging at single dendritic spines. Although this concentration of 4-AP has been shown to preferentially block native K^+^ channels containing Kv1 subunits, it will also affect some Kv4s (Coetzee et al., 1999; Gutman et al., 2003; Tseng, 1999). Superfusion of 4-AP (1 mM; in 64 spines, 8 dendrites in 8 cells) had no effect on the amplitude and duration of gluEPSPs (Fig. 2A). However, a higher concentration of 4-AP (5 mM; in 48 spines, 6 dendrites in 5 cells) prolonged the duration of gluEPSPs but had no effect on their amplitude (Fig. 2B). In a separate set of control experiments in which TTX was omitted from the perfusion solution we verified that 4-AP (1 and 5 mM) broadened action potentials elicited by supra-threshold depolarizing current steps (data not shown). To test the hypothesis that Kv4-containing K^+^ channels regulate gluEPSPs elicited by single spine activation we examined the effect of phrixotoxin-2, a peptide derived from the venom of the Chilean copper tarantula. Phrixotoxin-2 has been shown to block Kv4.2 and Kv4.3 channels but no other Kv subtypes expressed in heterologous systems (Diochot et al., 1999). In intact tissue, the application of phrixotoxin-2 (5 μM) decreased by 30% the peak of the A-type current recorded from sympathetic preganglionic neurons (Whyment et al., 2011), whereas in arcuate kisspeptin neurons this effect reached 50% (Mendonca et al., 2018). We used a similar concentration of phrixotoxin-2 in our experiments. Superfusion of phrixotoxin-2 (5 μM) increased the amplitude of gluEPSPs but the half width remained unchanged (Fig. 2C). Altogether, these data suggested that putative Kv4-containing K^+^ channels strongly depressed gluEPSPs generated by single spine stimulation.

Because changes in electrical membrane properties could in part explain the enhancing effect of K^+^ channel blockers on gluEPSPs, we analyzed how 4-AP and phrixotoxin-2 affected the membrane potential (V_m_), input resistance (R_in_) and membrane time-constant (τ_m_) of the recorded neurons. As shown in Fig. 2D, superfusion of phrixotoxin-2 (5 μM) had no effect on V_m_, R_in_ and τ_m_. Analyzing the changes over the entire data pool and for each pharmacological manipulation revealed that 4-AP (1 and 5 mM) depolarized L3 neurons, and that 4-AP (5 mM) significantly increased R_in_ and τ_m_ (Fig. 2D). We also tested the effect of the inorganic cation Ba^2+^ (Gasparini et al., 2007; Losonczy et al., 2008). When Ba^2+^ (150 μM) was added to the perfusion solution it substantially increased the amplitude, rise time, and half width of gluEPSPs (medians; peak amplitude: 0.87 *versus* 0.97 mV, *P* = 2E^−5^; half width: 14.56 *versus* 25.18 mV, *P* = 2E^−8^; Wilcoxon signed rank tests; data are from 56 spines, 9 dendrites in 9 cells, not shown). This effect was accompanied by membrane depolarization (ΔV_m_: 9.81 ± 3.34 mV) and a large increase in neuronal R_in_ (ΔR_in_: 121.45 ± 47.43%). Such dramatic changes in electrical membrane properties of L3 pyramidal neurons prevented a robust interpretation of the enhancing effect of Ba^2+^ on gluEPSPs. However, that phrixotoxin-2 enhanced gluEPSP amplitude with no other effect on V_m_, R_in_, and τ_m_, strengthened the finding that putative Kv4 channels exert a powerful brake on glutamatergic signals initiated at single spines.

### 2.3 Blocking putative Kv1 and Kv4 channels increases supralinear summation of inputs at clustered spines

**Fig. 3:**
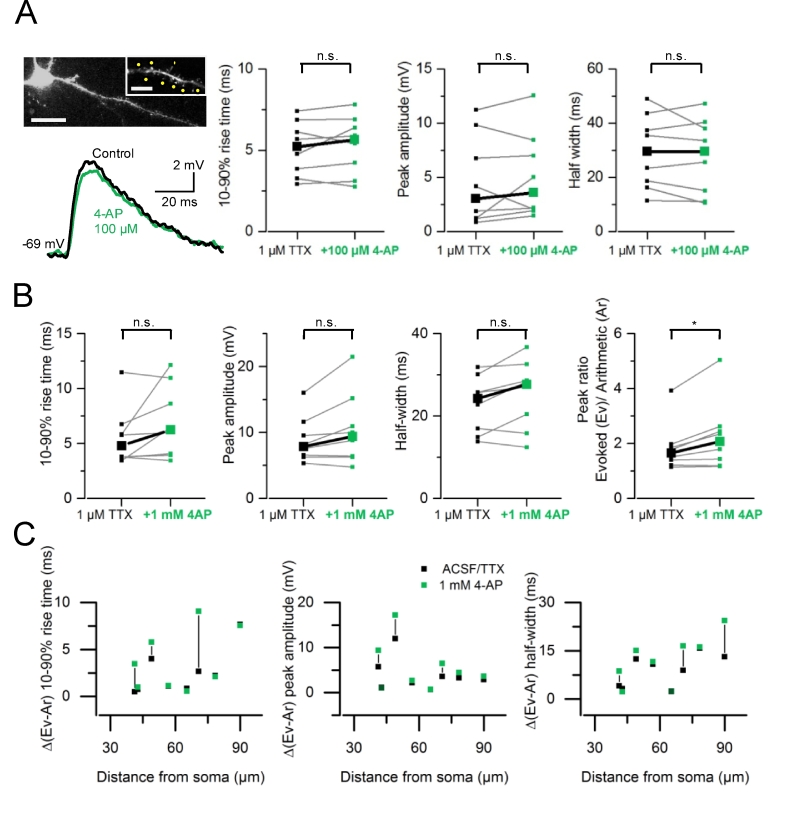
**Blocking A-type K**^**+**^**channels increases supralinear input summation. *A***, Left, maximum intensity projection of 2-photon images of a L3 pyramidal neuron filled with Alexa Fluor 594 (150 μM). The imaged dendrite and spines selected for uncaging (yellow spots) are shown at higher magnification (single image). Calibration bars: 20 μm and 5 μm (inset). Example traces show gluEPSPs (average of 10 consecutive trials) evoked by activation of a group of 8 spines, in control condition (1 μM TTX, black), and in the presence of 100 μM 4-AP (green). Right, summary graphs showing no change in gluEPSP rise time (medians; TTX: 5.23 *versus* 5.66 ms, *P* = 0.445, paired *t*-test), peak amplitude (TTX: 3.04 *versus* 3.61 mV, *P* = 0.92, Wilcoxon rank sum) or half width (TTX: 29.48 *versus* 29.6 ms, *P* = 0.36, paired *t*-test). Data are from 8 dendrites in 8 neurons. ***B***, Summary data showing no effect of 4-AP (1 mM) on gluEPSP rise time (medians; 4.79 *versus* 6.24 ms; paired *t*-test, *P* = 0.13), peak amplitude (7.86 *versus* 9.41 mV, *P* = 0.072), and half width (24.26 *versus* 27.68 ms, *P* = 0.059). The index of supralinearity (peak ratio) increases from 1.65 to 2.07 (Wilcoxon signed rank test, *P* = 0.016). ***C***, Deviation of gluEPSP properties (10–90% rise time, peak amplitude and half width) from the linear sum of underlying individual components, plotted against distance from the soma. All experiments were performed in the presence of TTX (1 μM) added to the perfusion solution. (For interpretation of the references to colour in this figure legend, the reader is referred to the web version of this article.)

The experiments performed in 4-AP (1 mM) suggest that a depolarization mimicking a synaptic input onto a single dendritic spine is insufficient to activate Kv1 channels. This prompts the question whether a larger EPSP and a prolonged rate of decay resulting from input summation can recruit putative Kv1s. We performed experiments using a low concentration of 4–AP (100 μM). At this concentration, 4-AP should mainly block Kv1 channels with no effect on Kv4s (Grissmer et al., 1994; Kirsch and Drewe, 1993). TTX (1 μM) and RuBi-glutamate (300 μM) were added to the control perfusion solution and a dendrite was chosen for imaging and uncaging. We recorded gluEPSPs in response to quasi-synchronous activation of 8 spines (with a 0.15 ms interval between stimulation of each spine) distributed over a region of ∼20–30 μm. (Fig. 3A). The application of 4–AP (100 μM) had no effect on such summed responses (n = 8 dendrites in 8 cells). The 10–90% rise time (medians ± S.D; TTX: 5.23 ± 1.67 *versus* 5.66 ± 1.8 ms, *P* = 0.445, paired *t*-test), peak amplitude (TTX: 3.04 ± 4.14 mV *versus* 3.61 ± 3.99 mV, *P* = 0.92, Wilcoxon rank sum) or half width (TTX: 29.48 ± 13.91 ms *versus* 29.60 ± 14.22 ms, *P* = 0.36, paired *t*-test) of gluEPSPs did not change (Fig. 3B). There was no effect of 4-AP (100 μM) on gluEPSPs elicited by the activation of each spine tested separately (rise time: *P* = 0.11; peak amplitude: *P* = 0.974; half width: *P* = 0.068; not shown). We then increased the concentration of 4-AP to 1 mM. Again, superfusion of 4-AP (1 mM) had no effect on the 10–90% rise time (TTX: 5.57 ± 2.69 ms *versus* 4-AP: 6.96 ± 3.31 ms, n = 8 dendrites in 8 cells; *P* = 0.134, paired *t*-test), peak amplitude (TTX: 8.89 ± 3.50 mV *versus* 4-AP: 10.48 ± 5.51 mV; *P* = 0.072, paired *t*-test) and half width (TTX: 22.75 ± 6.87 ms *versus* 4-AP: 25.24 ± 8.33 ms; *P* = 0.058, paired *t*-test) of summed gluEPSPs (Fig. 3C). Likewise, it did not affect gluEPSPs elicited by activation of single spines tested separately (see also Fig. 2A). However, the index of supralinearity was increased in 1 mM 4-AP (TTX: 1.85 ± 0.89 *versus* 4-AP: 2.26 ± 1.26; *P* = 0.015, Wilcoxon signed rank test; Fig. 3B). We did not find a preferential pattern of 4-AP-mediated modulation in supralinearity when plotting the changes in gluEPSP parameters against the dendritic path between the uncaging spot and the somatic recording site (Fig. 3C).

**Fig. 4:**
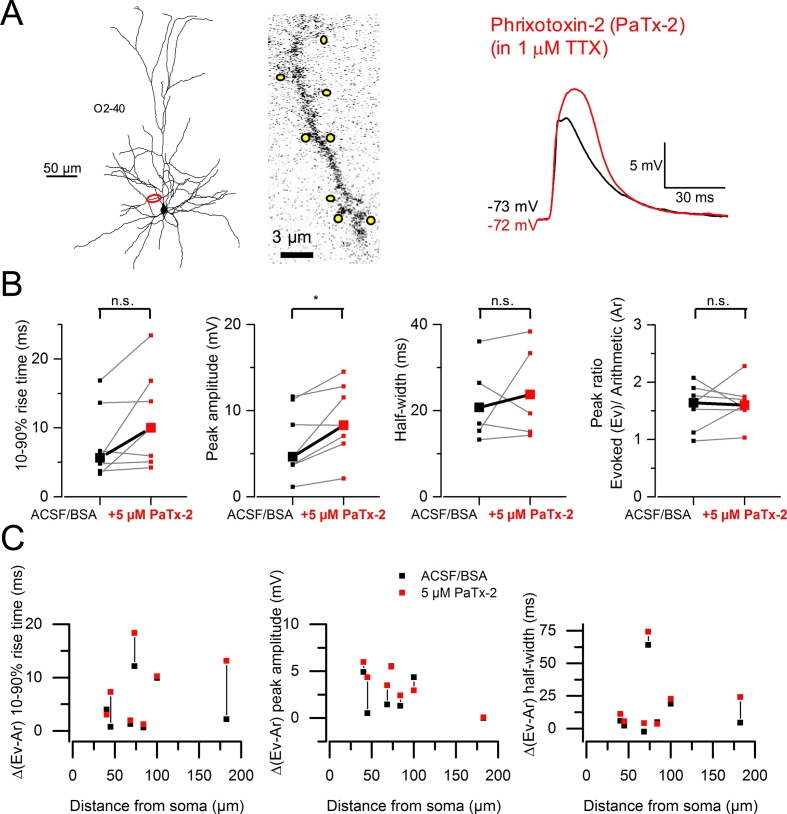
**Blocking putative Kv4 channels reveals additional components to summed gluEPSPs and increases supralinearity. *A***, Neurolucida reconstruction of a L3 pyramidal neuron with voltage recording and imaged dendrite. Yellow spots indicate 8 spines selected for group uncaging. O2 - 40: oblique, 2nd order, imaged at 40 μm. The block of putative Kv4 channels with phrixotoxin-2 (red trace) reveals additional gluEPSP components forming a delayed enhanced peak. ***B***, Summary graphs show increased gluEPSP amplitude in phrixotoxin-2, and no effect on the rise time and half width (medians; 10–90% rise time: 5.66 *versus* 10 ms, *P* = 0.089; peak amplitude: 4.66 *versus* 8.30 mV, *P* = 0.024; half width: 20.74 *versus* 23.77 ms, *P* = 0.264; paired *t*-tests, 7 dendrites in 6 cells). The peak ratio of evoked (Ev) to linear sum of individual components (Ar) does not change (supralinearity index; 1.64 *versus* 1.60; *P* = 0.705). *P < 0.05; n.s. non-significant. ***C***, Deviation of gluEPSP properties from the linear sum of individual components, plotted against distance from the soma. (For interpretation of the references to colour in this figure legend, the reader is referred to the web version of this article.)

We performed experiments using phrixotoxin-2 (5 μM; 7 dendrites in 6 cells) and found that it increased the amplitude of summed gluEPSPs (medians; ACSF/BSA: 6.36 ± 4.08 mV *versus* phrixotoxin-2: 8.94 ± 4.30 mV; *P* = 0.023, paired *t*-test) (Fig. 4A and B). The 10–90% rise time was not affected (7.81 ± 5.29 ms *versus* phrixotoxin-2: 11.33 ± 7.11 ms; *P* = 0.088, paired *t*-test), nor was the half width (control: 30.58 ± 25.34 ms *versus* phrixotoxin-2: 34.52 ± 29.15 ms; *P* = 0.264, paired *t*-test) (Fig. 4B). In contrast to the effect of 4-AP, phrixotoxin-2 increased the amplitude of gluEPSPs evoked by activation of single spines (see Fig. 2C). Consequently, the index of supralinearity of summed responses did not vary (control: 1.57 ± 0.40 *versus* phrixotoxin-2: 1.63 ± 0.37; *P* = 0.705, paired *t*-test). We found no indication that the effect of phrixotoxin-2 depended on distance along the dendritic path (Fig. 4C). These results demonstrated that putative Kv4 channels impede signal integration at clustered synapses. Kv1 channels, however, could be engaged in this phenomenon by reducing the amount of supralinearity of input summation.

**Fig. 5:**
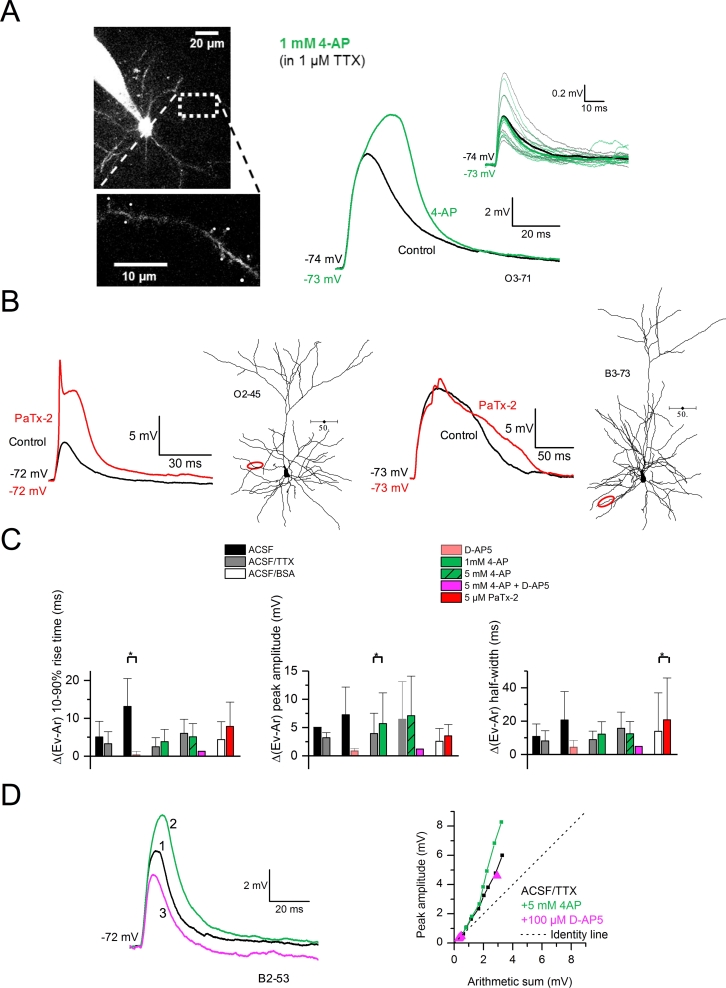
**Bi-directional modulation of dendritic integration via NMDA receptors and K**^**+**^**channels. *A***, Collapsed 2-photon Z-stack of a L3 pyramidal neuron filled with Alexa Fluor 594, with imaged dendrite shown as inset (3rd order oblique, 71 μm from the soma). Superfusion of 4-AP (1 mM) delays and enhances the peak of gluEPSPs elicited by group stimulation of 8 spines. Glu-EPSPs evoked by uncaging at single spines remain unaffected (top inset traces). Averaged gluEPSPs are shown as thick traces in both conditions (black: in TTX 1 μM, green: with 4-AP 1 mM). ***B,*** Neurolucida reconstructions of L3 pyramidal neurons with corresponding voltage recordings. Data are from 1 oblique dendrite (left) and 1 basal dendrite (right). O2 - 45: oblique, 2nd order, imaged 45 μm from the soma; B3 - 73: basal, 3rd order, imaged at 73 μm. The block of putative Kv4 channels (red traces) reveals dendritic spiking (left), or broadening of summed gluEPSPs (right). ***C,*** Bar charts quantifying the changes in supralinearity from control (black, grey, or white bars) to various drug conditions, as indicated. Data are from 2 to 8 neurons for each condition and are expressed as relative difference between the evoked gluEPSP and the linear sum of individual components. *P < 0.05, paired *t*-test. ***D***, Left, example of gluEPSP elicited by group stimulation of 8 spines (TTX, black trace). The summed gluEPSP is enhanced by 4-AP (green trace) and subsequently depressed by D-AP5 (magenta trace). Numbers indicate the order in which drugs were applied. Right, integration curves for this dendrite and for each drug condition. Dashed line represents a linear relation (identity line). B2 - 53: basal, 2nd order, imaged 53 μm from the soma. (For interpretation of the references to colour in this figure legend, the reader is referred to the web version of this article.)

Supralinear integration in layer 2/3 pyramidal cell dendrites depends on NMDA receptors, the recruitment of which is facilitated by activation of voltage-gated sodium channels and calcium channels (Branco and Hausser, 2011). Consistent with this finding, we found that the block of K^+^ currents led to a multiplicity of summed response shapes. Some cells (5 out of 8) showed a delayed and enhanced gluEPSP peak in 4-AP (Fig. 5A), whereas others exhibited dendritic spikes (4 out of 6) or gluEPSP broadening (2 out of 6) in the presence of phrixotoxin-2 (Fig. 5B). To test whether A-type K^+^ channels interact with NMDA receptors and voltage-gated conductances at clustered synaptic inputs we used again pharmacology (Fig. 5C). Switching from nACSF to a perfusion solution containing the NMDA receptor antagonist D(−)-2-amino-5-phosphonopentanoic acid (D-AP5, 100 μM) was accompanied by a reduction in the degree of supralinearity of summed gluEPSPs (10–90% rise time, nACSF: 13.13 ± 7.4 ms *versus* D-AP5: 0.35 ± 0.88 ms; n = 4 dendrites in 4 cells; *P* = *0.048*, paired *t*-test; Fig. 5C, left). The block of Na^+^ channels by TTX (1 μM) had no effect (10–90% rise time, *P* = 0.079; peak amplitude, *P* = 0.067; half width, *P* = 0.06; n = 3 dendrites in 3 cells; paired *t*-tests). In contrast, superfusion of 4-AP (1 mM) in the background of TTX enhanced supralinear integration of gluEPSPs (peak amplitude, TTX: 3.95 ± 3.37 mV *versus* +4-AP 1 mM: 5.54 ± 5.12 mV; *P* = 0.037, n = 8 dendrites in 8 cells; Fig. 5C, middle). A higher concentration of 4-AP (5 mM), however, did not cause significant changes (n = 6 dendrites in 6 cells). Lastly, phrixotoxin-induced facilitation in supralinearity was mainly manifested by changes in gluEPSP half width (ACSF/BSA: 13.77 ± 20.25 ms *versus* phrixotoxin-2: 19.21 ± 22.48 ms; *P* = 0.034, n = 7 dendrites in 6 cells; Fig. 5C, right). We also performed experiments where we first blocked K^+^ channels, and subsequently the NMDA receptors (n = 3 dendrites in 3 cells). As shown in the integration plots in Fig. 5D, the application of 4-AP (5 mM) enhanced gluEPSPs and increased the slope of the relation between gluEPSP amplitude and arithmetic sum of individual components. In contrast, the application of D-AP5 (100 μM) in the background of 4-AP reduced the steepness of this relationship. Altogether, these results highlighted the concerted role of A-type K^+^ channels and NMDA receptors in controlling the gain of signal integration in oblique and basal dendrites. I_A_ acted as a brake on supralinear input summation mediated by NMDA receptors during the synchronous activation of highly localized synapses.

### 2.4 A mechanistic description of I_A_ in a passive compartmental model supports close-to-homogenous distribution of A-type K^+^ conductance

**Fig. 6:**
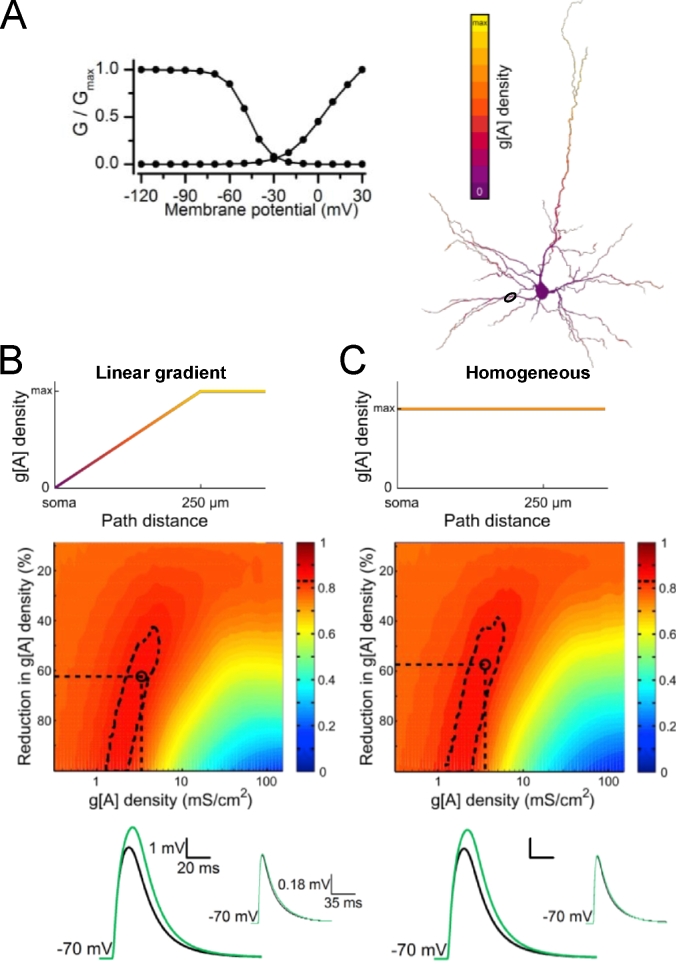
**Estimating the density of dendritic A-type conductance in a model that simulates increased supralinearity in 4-AP. *A***, Activation and inactivation curves of the transient A-type conductance incorporated with NMDA receptors in a passive and anatomically detailed model of a L3 pyramidal neuron (right). ***B***, The transient A-type conductance is distributed along a distance-dependent positive linear gradient capped at 250 μm from the soma. ***C***, The same conductance is distributed homogeneously. At the averaged experimental uncaging distance from the soma (80 μm, circle in neuron) a gluEPSP generated by activation of a single site and a gluEPSP generated by activation of 8 sites are simulated at a range of g[A] densities. For each modeled g[A] density, the degree of match between model and experiments for the combined relative changes in five parameters (amplitude and half width of single site and 8-site gluEPSPs, index of supralinearity) is evaluated across a range of reductions in g[A]. The resulting heat maps depict the interpolation of match values (color scale) across this parameter space compared to experimental results in 1 mM 4-AP. Regions of best match are defined as those that include the top 5% match values (dashed contours). Representative voltage traces result from simulations performed using a “control” g[A] density midway between the best match region boundaries and the corresponding midway “drug condition” g[A] density (black: control, green: 4-AP), at the coordinates indicated by black dashed lines and circle on heat maps. Simulated gluEPSPs for stimulation of 8 sites (large EPSP panels) and for underlying single site responses (upper right inset panels) are presented. (For interpretation of the references to colour in this figure legend, the reader is referred to the web version of this article.)

The pattern of distribution of A-type K^+^ channels along the fine dendrites of L3 pyramidal neurons is unknown. We took a modeling approach and asked whether I_A_ gradients were required to explain the set of changes in gluEPSP properties observed experimentally when bath-applying I_A_ blockers. To replicate the set of changes seen in 4-AP (1 mM) we created a computational model of a reconstructed L3 pyramidal neuron embedded with a transient A-type conductance (g[A], Fig. 6A). In one condition, we ran simulations where the g[A] density was set to a positive linear gradient along the dendritic path, caped at 250 μm from the soma (Fig. 6B). In an other condition, the g[A] density was distributed homogenously (Fig. 6C). We found that the largest value of match between model and experimental data was 0.88 for the gradient distribution and 0.87 for the homogenous distribution, defining regions of best match illustrated on heat maps (Fig. 6B and C). These regions extended over a range of g[A] densities set in pre-drug condition (1.30–5.41 mS/cm^2^ for gradient distribution; 1.29–5.93 mS/cm^2^ for homogenous distribution). An increase in amplitude and duration of summed gluEPSPs was replicated in the model as illustrated by simulated traces (Fig. 6B and C). Altogether, these finding suggest that a positive density gradient in g[A] along these dendrites is not required for explaining the set of experimental changes in gluEPSP properties in the presence of 4-AP.

## 3 Discussion

Our pharmacological analysis indicated that putative Kv4 channels depress glutamatergic signals elicited at single spines in basal and apical oblique dendrites of L3 pyramidal neurons. Putative Kv1 and Kv4 channels counteracted NMDA receptor contribution to summed gluEPSPs, hence reducing the gain of dendritic integration. In a computational model, we found that a linear density gradient of A-type conductance was not required for explaining the observed experimental changes in gluEPSP properties caused by K^+^ channel blockade.

Our results showing that phrixotoxin-2 enhanced the amplitude of gluEPSPs elicited by single spine excitation indicate that Kv4 channels impede small membrane depolarization and subsequent signal integration in L3 pyramidal neurons, confirming observations made in CA1 (Chen et al., 2006; Frick et al., 2003), in L5 pyramidal neurons (Carrasquillo et al., 2012), and in medial intercalated cells of the amygdala (Strobel et al., 2017). In hippocampal and cortical pyramidal neurons, Kv1 channels are blocked by a sub-millimolar concentration of 4-AP (Grissmer et al., 1994; Rasmusson et al., 1995), whereas Kv4 channels are blocked by 4-AP in the millimolar range (Norris and Nerbonne, 2010; Serodio et al., 1996). Because 4-AP (100 μM and 1 mM) had no effect on gluEPSPs evoked by uncaging at single spines, our data suggest that Kv1 channels might not be recruited by the depolarization resulting from the activation of a single synaptic input. The longer half width seen with a higher concentration of 4-AP (5 mM) could be explained by the 38% increase in τ_m_ although the amplitude of gluEPSPs did not change despite an overall similar increase in R_in_ (see Fig. 2D). Nevertheless, the 4-AP and phrixotoxin-2 data suggest that photostimulation at a single spine head generates a dendritic depolarization that is sufficiently large to activate Kv4 channels. Local EPSPs then propagate and attenuate along the dendritic path and are recorded as smaller somatic waveforms. In contrast, Kv1 channel activation might require a larger transmembrane voltage drop, such as seen during the synchronous activity of converging excitatory synaptic inputs (Rall, 1964). Besides the greater depolarization with multiple spine activation, a prolonged time course may also be important for activating the slower Kv1 channels to in turn, modulate the inputs. We found that 4-AP (100 μM and 1 mM) did not increase the amplitude and duration of summed gluEPSPs. The lack of statistical significance in the effect could have resulted from Kv channel inactivation in some of the studied dendrites, local saturation of dendritic potentials, or a combination of both. Further complication stems from the uncertainty about V_m_ in stimulated dendrites, and the fact that gluEPSPs attenuated from distinct branch divisions when propagating towards the soma. These confounding factors may have contributed to low statistical power in our analysis despite the reasonable number of repeats when studying summed responses (Fig. 4A and B; 16 dendrites). Notwithstanding this, our finding showing an increase in supralinearity in 4-AP (1 mM) indicates that putative Kv1 subunits (and some Kv4s) restrain the amplification of voltage components mediated by NMDA receptors. Because millimolar concentrations of 4-AP also blocked the persistent component of the K^+^ current expressed in neocortical pyramidal neurons, other Kv subunits might have contributed to the effect on supralinearity, notably Kv2 and Kv7 channels (Bishop et al., 2015; Guan et al., 2011; Pathak et al., 2016). Taken together, our phrixotoxin and 4-AP data support a role for Kv4 and Kv1 channels in reducing the amount of supralinearity of input summation at clustered synapses.

With regard to the pharmacological signature of the K^+^ channel blockers used here, we found that phrixotoxin-2 enhanced the peak amplitude of gluEPSPs whereas 4-AP (5 mM) prolonged the time-course. We interpret these effects as follows: Phrixotoxin-2 binds with high affinity at both closed and inactivated states of Kv4 channels where it disrupts the transmembrane movement of the voltage-sensing domain. Inhibition occurs as a result of a shift of the conductance-voltage relation and steady-state inactivation curve to more depolarized potentials (Chagot et al., 2004; Diochot et al., 1999). Thus, a maximum number of Kv4 channels will be bound to the toxin in baseline condition, mainly affecting the peak of gluEPSPs. In contrast, the 4-AP block is use-dependent and occurs preferentially at open Kv channels, via the intracellular side, with binding site accessibility and affinity controlled by channel gating (Choquet and Korn, 1992; Stephens et al., 1994). It is a slow process, which requires membrane depolarization, and most likely to prolong the rate of decay of gluEPSPs. Furthermore, 4-AP is a relatively less specific I_A_ blocker than is phrixotoxin-2, and at millimolar concentration it blocks a slow-activating and persistent K^+^ current, as well as other conductances that set the resting membrane potential and input resistance of L3 pyramidal neurons.

Blockade of A-type K^+^ channels is also known to drastically increase synaptic release of neurotransmitters. The depolarization of neighboring pyramidal neurons by 4-AP could in principle promote presynaptic changes, and contribute to the observed enhancement of gluEPSPs. We believe this was not the case. First, 4-AP (1 mM) did not affect gluEPSPs elicited by the activation of single spines despite significant depolarization and spike broadening. As for the high concentration of 4-AP (5 mM) we would expect the amplitude of gluPSPs to increase both as a result of enhanced neurotransmitter release and decreased input impedance, but there was no change. Secondly, the 4-AP experiments were conducted in the presence of TTX, which blocked action potential dependent neurotransmitter release, and reduced epileptiform activity secondary to K^+^ channel blockade. Thirdly, asynchronous glutamate release caused by K^+^ channel blockade in neighboring pyramidal neurons might as well increase the baseline occupancy of glutamate receptors in the recorded cell, and cause a minor reduction in the amplitude of gluEPSPs.

The functional diversity of Kv channels in L3 pyramidal cells could be further expanded by the patterned distribution of the A-type K^+^ conductance density as shown with dendritic patch-clamp in CA1 pyramidal cells and L5 neocortical neurons (Andrasfalvy et al., 2008; Gasparini et al., 2007; Harnett et al., 2013; Korngreen and Sakmann, 2000; Sun et al., 2011). We found that the effects of 4-AP and phrixotoxin-2 on supralinearity of synaptic integration did not depend on distance from the soma. Furthermore, our modeling could replicate the set of experimental changes in gluEPSP waveforms whether a positive linear gradient or a homogenous distribution of A-type conductance density was defined along the dendritic path. These results suggest that a steep gradient of A-type K^+^ channels might not be present in basal and oblique dendrites of L3 pyramidal cells. We also showed in the model that the region of best match to experimental 4-AP data extended over a range of reduction in g[A] density with mid-values greater than 60%. This level of block was in good agreement with the previously reported inhibition of I_A_ currents by 4-AP (1 mM) in L5 pyramidal neurons (Korngreen and Sakmann, 2000). In terms of channel numbers, and for the linear density distribution gradient of g[A], the region of best match to experimental data extended over a range of g[A] densities of 1.30–5.41 mS/cm^2^. Considering a mean single-channel conductance of 9 pS (Bekkers, 2000a) these conductance density values yielded 1–6 (midway 3) A-type K^+^ channels per μm^2^. For the homogenous g[A] density distribution, the model predicted 1–6 (midway 4) A-type K^+^ channels per μm^2^. These numbers were slightly smaller than the number of gold particles per μm^2^ counted in SDS-digested freeze-fracture replica-immunolabeling of Kv4.2 in radial oblique dendrites of CA1 pyramidal neurons (Kerti et al., 2012). However, they remained consistent with the number of Kv4.3 immunogold particles localized in spines and dendritic shaft in Purkinje cells (Otsu et al., 2014).

Our findings reveal that presumptive Kv1 and Kv4 channels restrain the window for summation of clustered glutamatergic inputs made onto basal and oblique dendrites of L3 pyramidal neurons. Kv4 channels regulate single synaptic inputs whereas Kv1-mediated modulation requires the activation of multiple inputs. Our results also lend support to the notion that Kv1 and Kv4 channels form separate functional entities (Carrasquillo et al., 2012; Foehring, 2012). Beyond, decreased expression and aberrant localization of Kv1.4, Kv4.2 and Kv4.3 have been reported in rodent models of epilepsy (Bernard et al., 2004; Lugo et al., 2008; Monaghan et al., 2008) and in a patient with temporal lobe epilepsy (Singh et al., 2006). Impaired closed-state inactivation of Kv4.2 channels caused by a *de novo* mutation in the KCND2 gene was recently discovered in identical twins affected by autism and intractable epilepsy (Lee et al., 2014). Finally, newly developed gene therapy where the K^+^ channel Kv1.1 was over-expressed in the epileptogenic focus successfully prevented the occurrence of electroencephalographic seizures (Wykes et al., 2012). A similar approach targeting both Kv1 and Kv4 channels could thus enhance the silencing of neocortical regions affected in paroxysmal excitability disorders.

## 4 Materials and methods

### 4.1 Animals

4-to-8 week-old C57BL/6J mice (Harlan Laboratories and Charles River or bred in-house) of both sexes were used. All the procedures were in accordance with the Home Office and UCL School of Pharmacy regulations regarding animal handling and experimental procedures (https://ethics.grad.ucl.ac.uk/).

### 4.2 Brain slice preparation

Mice were anesthetized with isoflurane (inhalatory, carrier O_2_) then pentobarbital sodium (80–150 mg/kg) injected intraperitoneally. In most cases mice were first transcardially perfused with ice-cold (∼4 °C) 50% sucrose-based ACSF, containing (in mM): NaCl (85), KCl (2.5), glucose (25), sucrose (75), NaHCO_3_ (24), NaH_2_PO_4_ (1.25), CaCl_2_ (0.5), MgCl_2_ (4). Following decapitation, mouse brains were quickly removed and placed into dissecting ACSF, the composition of which was identical to that of the transcardiac perfusion solution. Acute coronal cortical slices (300 μm thick) were obtained using a vibratome (VT1000S, Leica). For the storage of slices, the nACSF contained NaCl (126), KCl (2.5), glucose (25), NaHCO_3_ (24), NaH_2_PO_4_ (1.25), CaCl_2_ (2) and MgCl_2_ (2). Solutions were equilibrated with 95% O_2_/5% CO_2_ (pH 7.4, osmolality 315–325 mOsm.kg^−1^). Slices were incubated for 30 min at 34 °C in nACSF or 50% sucrose-based ACSF that was gradually replaced by nACSF in about 30 min using a peristaltic pump. Slices were then kept at room temperature in nACSF for up to 8 h, transferred to the recording chamber and superfused (2–3 ml/min) with nACSF at 30–32 °C. All solutions were prepared using water purchased from Fisher Scientific UK Ltd (Loughborough, UK).

### 4.3 Electrophysiological recordings

Whole-cell recordings were obtained from pyramidal neurons in L3 in putative cortical area V1. This cell type was chosen because of the relatively large number of oblique dendrites emerging from the main apical trunk. L3 pyramidal neurons were identified by Dodt-contrast imaging. Their morphological characteristics were revealed during live imaging followed by post-hoc analysis in fixed tissue. Patch pipettes were pulled from borosilicate glass capillaries (ID 0.86 mm, OD 1.50 mm with filament) with a Sutter P-2000 puller (Sutter Instruments). Pipettes of 3–5 MΩ resistance were filled with an internal solution containing (in mM): K gluconate (120), KCl (20), HEPES (10), NaCl (4), EGTA (0.05), MgATP (4), Na_2_GTP (0.45), phosphocreatinine-Na_2_ (14). Biocytin (0.2%w/v) and Alexa Fluor 594 (150 μM) were added to the internal solution (pH 7.25, osmolality 290–300 mOsm.kg^−1^). V_m_ was recorded using a Multiclamp 700B (Molecular Devices) amplifier, the NI USB-6259 board (National Instruments; data digitized at 20 kHz), and the NeuroMatic 2.6 software (http://www.neuromatic.thinkrandom.com; J. Rothman, UCL) running under Igor 6.2 (Wavemetrics). Following “break-in”, the access resistance was monitored in voltage-clamp mode and did not exceed 35 MΩ (typically below 20 MΩ). The bridge was then adjusted for all current-clamp recordings. A set of I—V relations and current injection protocols inspired by Toledo-Rodriguez et al. (2004) was applied to characterize the electrical membrane properties of the recorded neurons. R_in_ was measured as the steady-state voltage deflection in response to a hyperpolarizing current pulse (−30 pA, 300 ms) monitored throughout the experiment, and if it changed by more than 30% in control condition, the recording was discarded. Τ_m_ was determined by fitting a mono-exponential to the rising phase of the voltage traces used for determining R_in_. Supra-threshold depolarizing current steps (1 s) were also applied to characterize the firing pattern of the recorded cell. The pyramidal morphology of the recorded cell was then confirmed using two-photon imaging. During electrophysiological protocols, cells were held at −70 or −80 mV by DC current injection, except when monitoring V_m_ change during drug application. During uncaging protocols, current injection was performed by Multiclamp 700B function (dynamic, slow current injection, τ = 1 s). The effect of drugs on V_m_, τ_m_ and R_in_ was assessed for each cell by comparing averages of 10 trials of 300 ms current injections at 0.1 Hz, in each condition.

### 4.4 Two-photon imaging and one-photon glutamate uncaging

Two-photon laser-scanning microscopy was performed using a two-photon imaging system (Bruker Nano Surfaces, Middleton, USA), a titanium:sapphire MaiTai DeepSee ultrafast pulsed laser (Newport SpectraPhysics, UK), and a water-immersion objective (LUMPlanFL N 60× W, N.A. 1; Olympus UK). Alexa Fluor 594 included in intracellular solutions was excited at 870 nm. Glutamate was uncaged at 473 nm using a laser (Bruker Nano Surfaces, Middleton, USA) whose beam was directed into the microscope through optical fibers. A second set of galvanometers was used to control the targeting of the uncaging beam. In a typical experiment, a whole-cell recording was obtained from a putative L3 pyramidal neuron and the electrophysiological properties characterized. RuBi-glutamate (0.5 mM) was then bath-applied in nACSF (10 ml) and re-circulated via a peristaltic pump, at a perfusion rate of 2–3 ml/min, in dark or red light, for at least 15 min before the start of the uncaging. Alexa Fluor 594 was then visualized and dendritic segments that looked intact imaged. We selected dendritic regions that ran parallel to the slice surface for at least 20–50 μm at a depth of 40–60 μm. For each of these regions of interest, eight dendritic spines from the same branch were identified over a span of ∼20–30 μm. A locus near the head of each spine was then marked as uncaging site. The position, timing and intensity of laser pulses were controlled with TriggerSync (Bruker Nano Surfaces, Middleton, USA). Uncaging was performed using 0.2 ms duration laser pulses, and the evoked EPSPs (gluEPSP) were recorded at the soma. We designed two protocols to activate both single and multiple inputs within the same uncaging session: A short protocol, where 1 trial of group activation of 8 selected spines (with a 0.15 ms interval between stimulation of each spine) was followed 15 s later by 7 trials of uncaging at each of the spine (every 15 s, with a 700 ms interval between spines), yielding individual components of the EPSP. This was repeated 3 times in control condition, and 3 times in the presence of a blocker. A long protocol, made of 1 trial of group stimulation of an increasing number of spines (2, 3, 4, until 8) interleaved 15 s later with separate stimulation of each spine represented within the groups (with a 700 ms interval between spines). This was repeated 3 times in control and in the presence of a blocker to obtain integration curves for each condition.

To control for perturbing movements during long imaging experiments, a harp-shaped platinum wire was positioned at the surface of the slice. X-Y stage adjustments and objective refocusing to the location of dendritic spines were made in between stimulation protocols, in particular after a solution containing a pharmacological agent had been circulated. Successive responses elicited by uncaging at a particular spine (or group of spines) were very reproducible (see Fig. 1Bi) indicating that movements of the specimen did not contribute to pharmacological effects.

### 4.5 Drugs

4-aminopyridine (4-AP) was purchased from Sigma-Aldrich (St Louis, MO, USA). D-(−)-2-Amino-5-phosphonopentanoic acid (D-AP5) and TTX were obtained from Abcam Plc. (Cambridge, UK). Phrixotoxin-2 was purchased from Alomone Labs (Jerusalem, Israel) and diluted in 0.1% bovine serum albumin (BSA) solution. The dissolved phrixotoxin-2 was added to a perfusion solution containing 0.1% BSA. RuBi-glutamate was obtained from Tocris Cookson (Bristol, UK). To prevent dilution or contamination of re-circulated solutions, the perfusion tubing was drained when switching between drugs. Drugs were dissolved and kept as stock solutions at −20 °C, then diluted freshly before each experiment and applied for at least 10 min.

### 4.6 Data analysis

Electrophysiological data were analyzed using Igor Pro (Wavemetrics, Lake Oswego, USA), NeuroMatic (http://www.neuromatic.thinkrandom.com), Matlab 7.10 (MathWorks, Natick, USA) and Microsoft Excel 2010. Averaged waveforms were built from single sweeps aligned in time on the onset of the laser pulse and baseline-subtracted by the averaged voltage within 5 ms preceding the pulse. The 10–90% rise time, peak amplitude and half width of gluEPSPs were measured on such traces. To quantify the degree of supralinearity of input summation we excluded sweeps showing dendritic spikes. The peak amplitude, 10–90% rise time, and half width were measured on gluEPSP waveforms elicited by group stimulation of 8 spines (short protocol) or stimulation of 2 to all spines (long protocol). Similar measurements were performed on interleaved sweeps containing gluEPSPs evoked by activation of each spine involved in the summed response (individual components). GluEPSP supralinearity index was defined as the recorded EPSP peak over the peak of the linear sum of individual components. We also calculated the difference in peak amplitude, rise time and half width between the recorded gluEPSP and the linear sum of individual components Δ(Ev-Ar). Plotting the index of supralinearity against the age of the animals yielded no change postnatal days 22–48 (n = 20 mice; Pearson's coefficient R = 0.12, *P* = 0.6, ANOVA). For each selected dendrite where uncaging was performed, the peak of evoked gluEPSPs was plotted against the linear sum of individual components (integration curves, Fig. 5D) depicting changes in the gain of dendritic integration. Morphological measurements were performed using Neurolucida (MicroBrightField), NeuronStudio (Wearne et al., 2005), ImageJ (U. S. National Institutes of Health, Bethesda, Maryland) and Inkscape (www.inkscape.org). The path distance from the soma to the uncaging location was defined as the cumulative length of straight segments drawn onto the Z-projection of two-photon stacks. The branch order was determined from Neurolucida reconstructions or two-photon Z-stacks. Data from basal and oblique dendrites were pooled together and reported as mean ± S.D. unless otherwise indicated.

### 4.7 Statistical analysis

The Lilliefors test was used to inform data normality assumptions. One-way ANOVA test was used when comparing more than two samples. The Kruskal-Wallis test was used instead when the normality assumption could not be met for all groups. Similarly, paired comparisons were performed using either Student's *t*-test or Wilcoxon signed rank test, and unpaired comparisons were performed using either Student's *t*-test or Wilcoxon rank sum test. Statistical significance was considered when *P* < 0.05.

### 4.8 Histology and anatomical reconstructions

Following the experiment, slices were fixed overnight in 4% paraformaldehyde solution with 0.2% saturated picric acid solution and 0.1% gluteraldehyde in 0.1 M phosphate buffer (PB) at 4 °C. Slices were then washed and the fixative was exchanged with PB and slices subsequently processed with avidin-Texas Red (Vector Laboratories, CA, USA) to visualize biocytin (Sigma-Aldrich, St Louis, MO, USA). Briefly, slices were cryoprotected with sucrose and permeabilised by freeze-thawing over liquid nitrogen. Any endogenous peroxidase was blocked using 1% aqueous hydrogen peroxide and 1% sodium borohydride in PB to block free aldehyde groups. A goat serum block was performed with 10% normal goat serum prior to overnight incubation at 4 °C with ABC and Avidin Texas-Red (both Vector Laboratories, CA, USA). Slices were then mounted with Vectashield (Vector Laboratories, CA, USA) on a microscope slide. High-resolution Z-stacks of whole cells were acquired at 561 nm excitation with a Zeiss LSM 710 confocal microscope equipped with a 40× oil immersion objective (N.A. 1.3) to produce a 3-D tile-scan of the recovered cells. Slices were then sectioned to 60 μm and treated for HRP - DAB staining (Vectastain ABC kit) to produce a permanent record of neuronal morphology. Sections were osmocated then dehydrated with increasing concentration of ethanol, cleared with propylene and impregnated with resin (Durcupan) prior to mounting and curing. Neurolucida (MicroBrightField, Colchester, VT) was used to reconstruct the cells in 3-D both from confocal Z-stacks and HRP stained sections.

### 4.9 Computational modeling

The NEURON simulation environment (Hines and Carnevale, 1997) was used to perform simulations. The model was based on the detailed 3-D reconstruction (Neurolucida; Microbrightfield, Williston, VT) of a biocytin-filled L3 pyramidal neuron from one experiment. The spatial resolution of the digital morphological reconstruction was verified to comply with the ‘d_lambda rule’ (Carnevale and Hines, 2006). Passive parameters were R_a_ = 150 Ω cm, C_m_ = 1 μF/cm<sup>2</sup> in the soma, 2 μF/cm<sup>2</sup> in the dendrites accounting for spines, R_m_ = 6200 Ω cm^2^, resulting in a somatic R_in_ of 120 MΩ and a membrane time-constant of 10 ms. Simulations were performed at a resting V_m_ of −70 mV and a temperature of 32 °C. The responses from 8 identical synaptic sites separated by 2.4 μm were simulated on a dendritic section in random sequential combination with 0.35 ms inter-stimulus interval. AMPA receptor-mediated conductances were modeled as the difference between two exponential functions using the built-in class Exp2Syn, with time-constant and maximum conductance values that generated somatically recorded single-site responses comparable to those measured in experiments in control condition (1 mV peak amplitude, 16 ms width at half-amplitude e.g. τ_1_ = 0.33 ms, τ_2_ = 3.3 ms, g_max_ = 0.65 nS). NMDA receptor-mediated conductances were modeled using the implementation of a 10 state kinetic model from Kampa et al. (2004) by Branco et al. (2010). The maximal NMDA conductance was adjusted together with the maximal AMPA conductance to preserve the properties of somatically recorded gluEPSPs evoked by uncaging at single spines, while providing levels of supralinearity comparable to that seen in experiments when uncaging at 8 sites (effective maximal NMDA conductance of 1.33 nS for AMPA to NMDA ratio of 1:3). The mechanism used for the A-type conductance is available from ModelDB at http://senselab.med.yale.edu/modeldb with accession number 3660, implemented by M. Migliore for superficial neocortical pyramidal neurons based on a study by Zhou and Hablitz (1996). Activation and inactivation kinetics were left unchanged, whereas steady-state voltage at half activation and inactivation, and corresponding slopes were modified to match the average values in Zhou and Hablitz (1996): V_1/2 activ_ = 0.5 mV, slope_activ_ = −12.2 mV; V_1/2 inact_ = −47.4 mV, slope_inact_ = 7.2 mV, for Boltzmann functions in the form:

$$\frac{G}{G_{max}}=\frac{1}{1+e^{\left(\frac{\;V-V_{1/2}}{slope}\right)}}$$

where G_max_ is the maximal conductance, and G the conductance at a particular steady-state potential V.

Somatic V_m_ was stabilized at −70 mV with increasing densities of A-type conductance by current injection. Match between model and experimental data was calculated using the following formula:

$$Match=\frac{1}{1+\sqrt{\sum_{k}{\left(\frac{Xm_{k}-Xe_{k}}{Xe_{k}}\right)}^{2}}}$$

where *k* is an index of the five gluEPSP parameters considered for the simulations (peak amplitude and half width of gluEPSPs evoked by uncaging at single spines and eight spines, and index of supralinearity calculated as the ratio of amplitude of evoked to summed gluEPSPs), *Xe* is the average relative change between drug and control condition detected experimentally, and *Xm* is the same change measured on simulated traces, in the model.

## Funding

This work was supported by the Medical Research Council UK (Project grant number G1000629) and partly by the Human Brain Project (HBP-604102 grant) awarded to Alex M Thomson.

## Conflicts of interest

The authors declare no financial and non-financial competing interests.

## Authors contribution

ABi, ABr, and AR designed the study. ABi and AR performed the imaging, uncaging, and electrophysiology. ABr performed the computational modeling. JF did the histological analysis and anatomical reconstructions. ABi, ABr and AR analyzed the electrophysiological and imaging data. AR wrote the manuscript, which was then revised by all the authors.

## References

[bib1] Andrasfalvy B.K., Makara J.K., Johnston D., Magee J.C. (2008). Altered synaptic and non-synaptic properties of CA1 pyramidal neurons in Kv4.2 knockout mice. J. Physiol..

[bib2] Araya R. (2014). Input transformation by dendritic spines of pyramidal neurons. Front. Neuroanat..

[bib3] Bekkers J.M. (2000). Distribution and activation of voltage-gated potassium channels in cell-attached and outside-out patches from large layer 5 cortical pyramidal neurons of the rat. J. Physiol..

[bib4] Bekkers J.M. (2000). Properties of voltage-gated potassium currents in nucleated patches from large layer 5 cortical pyramidal neurons of the rat. J. Physiol..

[bib5] Bekkers J.M., Delaney A.J. (2001). Modulation of excitability by alpha-dendrotoxin-sensitive potassium channels in neocortical pyramidal neurons. J. Neurosci..

[bib6] Bernard C., Anderson A., Becker A., Poolos N.P., Beck H., Johnston D. (2004). Acquired dendritic channelopathy in temporal lobe epilepsy. Science.

[bib7] Bishop H.I., Guan D., Bocksteins E., Parajuli L.K., Murray K.D., Cobb M.M., Misonou H., Zito K., Foehring R.C., Trimmer J.S. (2015). Distinct cell- and layer-specific expression patterns and independent regulation of Kv2 channel subtypes in cortical pyramidal neurons. J. Neurosci..

[bib8] Branco T., Clark B.A., Hausser M. (2010). Dendritic discrimination of temporal input sequences in cortical neurons. Science.

[bib9] Branco T., Hausser M. (2010). The single dendritic branch as a fundamental functional unit in the nervous system. Curr. Opin. Neurobiol..

[bib10] Branco T., Hausser M. (2011). Synaptic integration gradients in single cortical pyramidal cell dendrites. Neuron.

[bib11] Burkhalter A., Gonchar Y., Mellor R.L., Nerbonne J.M. (2006). Differential expression of I(A) channel subunits Kv4.2 and Kv4.3 in mouse visual cortical neurons and synapses. J. Neurosci..

[bib12] Cai X., Liang C.W., Muralidharan S., Kao J.P., Tang C.M., Thompson S.M. (2004). Unique roles of SK and Kv4.2 potassium channels in dendritic integration. Neuron.

[bib13] Carnevale N.T., Hines M. (2006). The NEURON Book.

[bib14] Carrasquillo Y., Burkhalter A., Nerbonne J.M. (2012). A-type K+ channels encoded by Kv4.2, Kv4.3 and Kv1.4 differentially regulate intrinsic excitability of cortical pyramidal neurons. J. Physiol..

[bib15] Chagot B., Escoubas P., Villegas E., Bernard C., Ferrat G., Corzo G., Lazdunski M., Darbon H. (2004). Solution structure of Phrixotoxin 1, a specific peptide inhibitor of Kv4 potassium channels from the venom of the theraphosid spider Phrixotrichus auratus. Protein Sci..

[bib16] Chen X., Leischner U., Rochefort N.L., Nelken I., Konnerth A. (2011). Functional mapping of single spines in cortical neurons in vivo. Nature.

[bib17] Chen X., Yuan L.L., Zhao C., Birnbaum S.G., Frick A., Jung W.E., Schwarz T.L., Sweatt J.D., Johnston D. (2006). Deletion of Kv4.2 gene eliminates dendritic A-type K+ current and enhances induction of long-term potentiation in hippocampal CA1 pyramidal neurons. J. Neurosci..

[bib18] Choquet D., Korn H. (1992). Mechanism of 4-aminopyridine action on voltage-gated potassium channels in lymphocytes. J. Gen. Physiol..

[bib19] Coetzee W.A., Amarillo Y., Chiu J., Chow A., Lau D., McCormack T., Moreno H., Nadal M.S., Ozaita A., Pountney D., Saganich M., Vega-Saenz de Miera E., Rudy B. (1999). Molecular diversity of K+ channels. Ann. N. Y. Acad. Sci..

[bib20] Diochot S., Drici M.D., Moinier D., Fink M., Lazdunski M. (1999). Effects of phrixotoxins on the Kv4 family of potassium channels and implications for the role of Ito1 in cardiac electrogenesis. Br. J. Pharmacol..

[bib21] Foehring R.C. (2012). What does type A mean to a pyramidal cell?. J. Physiol..

[bib22] Foehring R.C., Surmeier D.J. (1993). Voltage-gated potassium currents in acutely dissociated rat cortical neurons. J. Neurophysiol..

[bib23] Frick A., Magee J., Koester H.J., Migliore M., Johnston D. (2003). Normalization of Ca2+ signals by small oblique dendrites of CA1 pyramidal neurons. J. Neurosci..

[bib24] Gasparini S., Losonczy A., Chen X., Johnston D., Magee J.C. (2007). Associative pairing enhances action potential back-propagation in radial oblique branches of CA1 pyramidal neurons. J. Physiol..

[bib25] Golding N.L., Jung H.Y., Mickus T., Spruston N. (1999). Dendritic calcium spike initiation and repolarization are controlled by distinct potassium channel subtypes in CA1 pyramidal neurons. J. Neurosci..

[bib26] Grissmer S., Nguyen A.N., Aiyar J., Hanson D.C., Mather R.J., Gutman G.A., Karmilowicz M.J., Auperin D.D., Chandy K.G. (1994). Pharmacological characterization of five cloned voltage-gated K+ channels, types Kv1.1, 1.2, 1.3, 1.5, and 3.1, stably expressed in mammalian cell lines. Mol. Pharmacol..

[bib27] Guan D., Armstrong W.E., Foehring R.C. (2015). Electrophysiological properties of genetically identified subtypes of layer 5 neocortical pyramidal neurons: Ca(2)(+) dependence and differential modulation by norepinephrine. J. Neurophysiol..

[bib28] Guan D., Horton L.R., Armstrong W.E., Foehring R.C. (2011). Postnatal development of A-type and Kv1- and Kv2-mediated potassium channel currents in neocortical pyramidal neurons. J. Neurophysiol..

[bib29] Guan D., Lee J.C., Higgs M.H., Spain W.J., Foehring R.C. (2007). Functional roles of Kv1 channels in neocortical pyramidal neurons. J. Neurophysiol..

[bib30] Guan D., Lee J.C., Tkatch T., Surmeier D.J., Armstrong W.E., Foehring R.C. (2006). Expression and biophysical properties of Kv1 channels in supragranular neocortical pyramidal neurones. J. Physiol..

[bib31] Gutman G.A., Chandy K.G., Adelman J.P., Aiyar J., Bayliss D.A., Clapham D.E., Covarriubias M., Desir G.V., Furuichi K., Ganetzky B., Garcia M.L., Grissmer S., Jan L.Y., Karschin A., Kim D., Kuperschmidt S., Kurachi Y., Lazdunski M., Lesage F., Lester H.A., McKinnon D., Nichols C.G., O'Kelly I., Robbins J., Robertson G.A., Rudy B., Sanguinetti M., Seino S., Stuehmer W., Tamkun M.M., Vandenberg C.A., Wei A., Wulff H., Wymore R.S., International Union of, P (2003). International Union of Pharmacology. XLI. Compendium of voltage-gated ion channels: potassium channels. Pharmacol. Rev..

[bib32] Harnett M.T., Xu N.L., Magee J.C., Williams S.R. (2013). Potassium channels control the interaction between active dendritic integration compartments in layer 5 cortical pyramidal neurons. Neuron.

[bib33] Hille B. (2001). Ion Channels of Excitable Membranes.

[bib34] Hines M.L., Carnevale N.T. (1997). The NEURON simulation environment. Neural Comput..

[bib35] Hoffman D.A., Magee J.C., Colbert C.M., Johnston D. (1997). K+ channel regulation of signal propagation in dendrites of hippocampal pyramidal neurons. Nature.

[bib36] Jerng H.H., Pfaffinger P.J., Covarrubias M. (2004). Molecular physiology and modulation of somatodendritic A-type potassium channels. Mol. Cell. Neurosci..

[bib37] Juiz J.M., Lujan R., Dominguez del Toro E., Fuentes V., Ballesta J.J., Criado M. (2000). Subcellular compartmentalization of a potassium channel (Kv1.4): preferential distribution in dendrites and dendritic spines of neurons in the dorsal cochlear nucleus. Eur. J. Neurosci..

[bib38] Kampa B.M., Clements J., Jonas P., Stuart G.J. (2004). Kinetics of Mg2+ unblock of NMDA receptors: implications for spike-timing dependent synaptic plasticity. J. Physiol..

[bib39] Kang J., Huguenard J.R., Prince D.A. (2000). Voltage-gated potassium channels activated during action potentials in layer V neocortical pyramidal neurons. J. Neurophysiol..

[bib40] Katz Y., Menon V., Nicholson D.A., Geinisman Y., Kath W.L., Spruston N. (2009). Synapse distribution suggests a two-stage model of dendritic integration in CA1 pyramidal neurons. Neuron.

[bib41] Kerti K., Lorincz A., Nusser Z. (2012). Unique somato-dendritic distribution pattern of Kv4.2 channels on hippocampal CA1 pyramidal cells. Eur. J. Neurosci..

[bib42] Kim J., Jung S.C., Clemens A.M., Petralia R.S., Hoffman D.A. (2007). Regulation of dendritic excitability by activity-dependent trafficking of the A-type K+ channel subunit Kv4.2 in hippocampal neurons. Neuron.

[bib43] Kirsch G.E., Drewe J.A. (1993). Gating-dependent mechanism of 4-aminopyridine block in two related potassium channels. J. Gen. Physiol..

[bib44] Korngreen A., Sakmann B. (2000). Voltage-gated K+ channels in layer 5 neocortical pyramidal neurones from young rats: subtypes and gradients. J. Physiol..

[bib45] Korogod S., Tyc-Dumont S. (2009). Electrical Dynamics of the Dendritic Space.

[bib46] Krueppel R., Remy S., Beck H. (2011). Dendritic integration in hippocampal dentate granule cells. Neuron.

[bib47] Lee H., Lin M.C., Kornblum H.I., Papazian D.M., Nelson S.F. (2014). Exome sequencing identifies de novo gain of function missense mutation in KCND2 in identical twins with autism and seizures that slows potassium channel inactivation. Hum. Mol. Genet..

[bib48] Locke R.E., Nerbonne J.M. (1997). Role of voltage-gated K+ currents in mediating the regular-spiking phenotype of callosal-projecting rat visual cortical neurons. J. Neurophysiol..

[bib49] Losonczy A., Magee J.C. (2006). Integrative properties of radial oblique dendrites in hippocampal CA1 pyramidal neurons. Neuron.

[bib50] Losonczy A., Makara J.K., Magee J.C. (2008). Compartmentalized dendritic plasticity and input feature storage in neurons. Nature.

[bib51] Lugo J.N., Barnwell L.F., Ren Y., Lee W.L., Johnston L.D., Kim R., Hrachovy R.A., Sweatt J.D., Anderson A.E. (2008). Altered phosphorylation and localization of the A-type channel, Kv4.2 in status epilepticus. J. Neurochem..

[bib52] Magee J.C. (2000). Dendritic integration of excitatory synaptic input. Nat. Rev. Neurosci..

[bib53] Major G., Larkum M.E., Schiller J. (2013). Active properties of neocortical pyramidal neuron dendrites. Annu. Rev. Neurosci..

[bib54] Makara J.K., Losonczy A., Wen Q., Magee J.C. (2009). Experience-dependent compartmentalized dendritic plasticity in rat hippocampal CA1 pyramidal neurons. Nat. Neurosci..

[bib55] Marionneau C., LeDuc R.D., Rohrs H.W., Link A.J., Townsend R.R., Nerbonne J.M. (2009). Proteomic analyses of native brain K(V)4.2 channel complexes. Channels (Austin).

[bib56] Mendonca P.R.F., Kyle V., Yeo S.H., Colledge W.H., Robinson H.P.C. (2018). Kv4.2 channel activity controls intrinsic firing dynamics of arcuate kisspeptin neurons. J. Physiol..

[bib57] Monaghan M.M., Menegola M., Vacher H., Rhodes K.J., Trimmer J.S. (2008). Altered expression and localization of hippocampal A-type potassium channel subunits in the pilocarpine-induced model of temporal lobe epilepsy. Neuroscience.

[bib58] Norris A.J., Nerbonne J.M. (2010). Molecular dissection of I(A) in cortical pyramidal neurons reveals three distinct components encoded by Kv4.2, Kv4.3, and Kv1.4 alpha-subunits. J. Neurosci..

[bib59] Otsu Y., Marcaggi P., Feltz A., Isope P., Kollo M., Nusser Z., Mathieu B., Kano M., Tsujita M., Sakimura K., Dieudonne S. (2014). Activity-dependent gating of calcium spikes by A-type K+ channels controls climbing fiber signaling in Purkinje cell dendrites. Neuron.

[bib60] Ovsepian S.V., Steuber V., Le Berre M., O'Hara L., O'Leary V.B., Dolly J.O. (2013). A defined heteromeric KV1 channel stabilizes the intrinsic pacemaking and regulates the output of deep cerebellar nuclear neurons to thalamic targets. J. Physiol..

[bib61] Pathak D., Guan D., Foehring R.C. (2016). Roles of specific Kv channel types in repolarization of the action potential in genetically identified subclasses of pyramidal neurons in mouse neocortex. J. Neurophysiol..

[bib62] Rall W. (1964). Theoretical Significance of Dendritic Trees for Neuronal Input-output Relations.

[bib63] Rasmusson R.L., Zhang Y., Campbell D.L., Comer M.B., Castellino R.C., Liu S., Strauss H.C. (1995). Bi-stable block by 4-aminopyridine of a transient K+ channel (Kv1.4) cloned from ferret ventricle and expressed in Xenopus oocytes. J. Physiol..

[bib64] Serodio P., Vega-Saenz de Miera E., Rudy B. (1996). Cloning of a novel component of A-type K+ channels operating at subthreshold potentials with unique expression in heart and brain. J. Neurophysiol..

[bib65] Singh B., Ogiwara I., Kaneda M., Tokonami N., Mazaki E., Baba K., Matsuda K., Inoue Y., Yamakawa K. (2006). A Kv4.2 truncation mutation in a patient with temporal lobe epilepsy. Neurobiol. Dis..

[bib66] Spruston N. (2008). Pyramidal neurons: dendritic structure and synaptic integration. Nat. Rev. Neurosci..

[bib67] Stephens G.J., Garratt J.C., Robertson B., Owen D.G. (1994). On the mechanism of 4-aminopyridine action on the cloned mouse brain potassium channel mKv1.1. J. Physiol..

[bib68] Strobel C., Sullivan R.K.P., Stratton P., Sah P. (2017). Calcium signalling in medial intercalated cell dendrites and spines. J. Physiol..

[bib69] Stuart G.J., Spruston N. (2015). Dendritic integration: 60 years of progress. Nat. Neurosci..

[bib70] Sun W., Maffie J.K., Lin L., Petralia R.S., Rudy B., Hoffman D.A. (2011). DPP6 establishes the A-type K(+) current gradient critical for the regulation of dendritic excitability in CA1 hippocampal neurons. Neuron.

[bib71] Toledo-Rodriguez M., Blumenfeld B., Wu C., Luo J., Attali B., Goodman P., Markram H. (2004). Correlation maps allow neuronal electrical properties to be predicted from single-cell gene expression profiles in rat neocortex. Cereb Cortex.

[bib72] Tseng G.N. (1999). Different state dependencies of 4-aminopyridine binding to rKv1.4 and rKv4.2: role of the cytoplasmic halves of the fifth and sixth transmembrane segments. J Pharmacol Exp Ther.

[bib73] Wang K., Lin M.T., Adelman J.P., Maylie J. (2014). Distinct Ca2+ sources in dendritic spines of hippocampal CA1 neurons couple to SK and Kv4 channels. Neuron.

[bib74] Wearne S.L., Rodriguez A., Ehlenberger D.B., Rocher A.B., Henderson S.C., Hof P.R. (2005). New techniques for imaging, digitization and analysis of three-dimensional neural morphology on multiple scales. Neuroscience.

[bib75] Whyment A.D., Coderre E., Wilson J.M., Renaud L.P., O'Hare E., Spanswick D. (2011). Electrophysiological, pharmacological and molecular profile of the transient outward rectifying conductance in rat sympathetic preganglionic neurons in vitro. Neuroscience.

[bib76] Williams S.R., Stuart G.J. (2002). Dependence of EPSP efficacy on synapse location in neocortical pyramidal neurons. Science.

[bib77] Wykes R.C., Heeroma J.H., Mantoan L., Zheng K., MacDonald D.C., Deisseroth K., Hashemi K.S., Walker M.C., Schorge S., Kullmann D.M. (2012). Optogenetic and potassium channel gene therapy in a rodent model of focal neocortical epilepsy. Sci. Transl. Med..

[bib78] Zhou F.M., Hablitz J.J. (1996). Layer I neurons of the rat neocortex. II. Voltage-dependent outward currents. J. Neurophysiol..

